# Plasma Cytokeratin-18 Fragment Level Reflects the Metabolic Phenotype in Obesity

**DOI:** 10.3390/biom13040675

**Published:** 2023-04-14

**Authors:** Joanna Goralska, Urszula Razny, Anna Gruca, Anna Zdzienicka, Agnieszka Micek, Aldona Dembinska-Kiec, Bogdan Solnica, Malgorzata Malczewska-Malec

**Affiliations:** 1 Department of Clinical Biochemistry, Jagiellonian University Medical College, Skawinska 8, 31-066 Krakow, Poland; 2 Institute of Nursing and Midwifery, Jagiellonian University Medical College; Michałowskiego 12, 31-126 Krakow, Poland

**Keywords:** cytokeratin-18, CK-18, fatty liver, obesity, FGF-21

## Abstract

There is growing interest in the non-invasive identification and monitoring of the outcome of liver damage in obese patients. Plasma cytokeratin-18 (CK-18) fragment levels correlate with the magnitude of hepatocyte apoptosis and have recently been proposed to independently predict the presence of non-alcoholic steatohepatitis (NASH). The aim of the study was to analyze the associations of CK-18 with obesity and related complications: insulin resistance, impaired lipid metabolism and the secretion of hepatokines, adipokines and pro-inflammatory cytokines. The study involved 151 overweight and obese patients (BMI 25–40), without diabetes, dyslipidemia or apparent liver disease. Liver function was assessed based on alanine aminotransferase (ALT), gamma-glutamyl transferase (GGT) and the fatty liver index (FLI). CK-18 M30 plasma levels, FGF-21, FGF-19 and cytokines were determined by ELISA. CK-18 values >150 U/l were accompanied by high ALT, GGT and FLI, insulin resistance, postprandial hypertriglyceridemia, elevated FGF-21 and MCP-1 and decreased adiponectin. ALT activity was the strongest independent factor influencing high CK-18 plasma levels, even after an adjustment for age, sex and BMI [β coefficient (95%CI): 0.40 (0.19–0.61)]. In conclusion, the applied CK-18 cut-off point at 150 U/l allows to distinguish between two metabolic phenotypes in obesity.

## 1. Introduction

There is growing interest in the non-invasive identification and monitoring of the outcome of liver damage in obese patients [1,2,3]. Non-alcoholic fatty liver disease (NAFLD) was recently termed metabolic dysfunction-associated fatty liver disease (MAFLD) which more closely implicates the presence of overweight/obesity and metabolic dysregulation as essential pathogenic factors of this chronic condition [4,5]. The intracellular accumulation of fat at the initial stage may evolve into non-alcoholic steatohepatitis, accompanied by inflammation, fibrosis and apoptosis. The progression of MAFLD involves hepatocyte swelling and alterations in proteins that build the cytoskeleton. An analysis of cytoskeleton proteins may provide some information on the molecular basis of MAFLD pathogenesis and improve the NAFLD diagnosis and staging, especially in terms of the transition from benign simple steatosis to irreversible steatohepatitis [6].

Metabolic risk factors, such as increased waist circumference, hypertension, impaired fasting glucose or diabetes, elevated serum triglycerides and decreased HDL cholesterol, increase the risk of more advanced MAFLD. Although the need for fatty liver screening in the general population has been questioned given the high direct and indirect costs of testing, the risk of liver biopsy and the low predictive value of non-invasive tests, there is a need for the identification of the progressive form of NAFLD, particularly when associated with advanced fibrosis [7]. According to EASL-AESD-EASO guidelines, the diagnosis of steatosis should be documented by imaging, although the quantification of fat content is not of interest. However, whenever imaging tools are not available or feasible, serum biomarkers and scores are an acceptable alternative for the diagnosis of steatosis. The best validated steatosis scores—in the general population, as well as in obese subjects—are the fatty liver index (FLI), hepatic steatosis test (HSI), the NAFLD screening score and SteatoTest. The FLI is the prevalent biomarker panel consisting of body mass index, waist circumference, triglycerides and gamma-glutamyl transferase for identifying NAFLD, with an AUROC of 0.84 [8]. The development and use of non-invasive fibrosis tests has reduced the need for liver biopsies. However, the FLI, similar to other scores, poorly distinguishes moderate-to-severe steatosis from mild steatosis; thus, some data suggest that the combination of elastography (FibroScan) and serum markers performs better than either method alone [9].

For the assessment of fibrosis stage, the NAFLD fibrosis score (NFS), enhanced liver fibrosis (ELF), FibroTest and fibrosis 4 calculator (FIB-4) have acceptable diagnostic accuracy [10]. According to recommendations, these serum fibrosis markers are of special interest in patients with steatosis and normal liver enzymes (ALT, AST, GGT) who may still be at a risk of advanced fibrosis [7]. A recent update of guidelines indicates that the non-invasive fibrosis test should be used for ruling out rather than diagnosing advanced fibrosis in low-prevalence populations, as well as being used preferentially in patients with metabolic risk factors [11]. In routine investigations, the simple non-invasive fibrosis score FIB-4, apart from ALT, AST and platelet counts, in individuals with metabolic risk factors or the harmful use of alcohol, is recommended in order to improve risk stratification. Other non-invasive emerging biomarkers aimed to differentiate NASH from simple steatosis include proinflammatory cytokines (e.g., CXCL10, TNFα, IL-8), iron metabolism markers (e.g., serum iron, ferritin) and hormones (e.g., FGF21, adiponectin, leptin, resistin) [12]. Noteworthy, better predictive values are obtained when FGF21 was combined with cytokeratin-18 (PPV 82%, NPV 74%), as well as when adiponectin was combined with cytokeratin-18 and IL-8 (AUROC 0.90) [13]. A novel biomarker panel, the NASH ClinLipMet score, also includes a genetic factor, the PNPLA3 genotype, in addition to AST, fasting insulin and metabolic-syndrome-based factors, but is not useful in clinical practice [14]. Another interesting method, based on breath volatile organic compound determination, analyzes the exhaled biomarkers of oxidative stress and inflammation related to liver diseases [15]. The development of molecular techniques allows for the study of new biomarkers of liver damage and fibrosis, such as microRNAs [16,17]. Likewise, increased mitochondrial mass along with higher levels of mitochondrial cell-free circulating DNA (mt-ccf) may reflect D-loop mtDNA mutations causing the loss of mitochondrial flexibility during NAFLD progression to NASH [18]. 

Although some emerging biomarkers of NASH have been investigated, they need to be validated and, as for now, liver biopsy, despite some limitations, is the only procedure that reliably differentiates simple steatosis from NASH, presenting not only steatosis but also ballooning and lobular inflammation. However, it is worth noting that additional histological features of NASH include apoptotic bodies and Mallory-Denk bodies (MDBs), significantly associated with alterations in the cytoskeleton, mainly reorganization of the cytokeratin system, including the heterodimer CK8/CK18 network [19].

Three major types of filaments make up the cytoskeleton: actin filaments, microtubules and intermediate filaments. Unlike actin filaments or microtubules, intermediate filaments are diverse in protein composition, depending on distinct cell types; thus, the analysis of intermediate filament proteins in the bloodstream may provide some information about their origin. In the liver, these proteins include vimentin, desmin, nestin and glial fibrillary acidic protein, expressed in hepatic stellate cells, as well as cytokeratin-8 and cytokeratin-18, forming heterodimers expressed in hepatocytes [20]. Due to the simple cytokeratin expression pattern, hepatocytes are particularly sensitive to alterations in cytokeratin architecture, and changes in cytokeratin-18 (CK-18) may indicate liver injury [4]. Nevertheless, cytokeratins represents the largest subfamily (>50 functional genes) of intermediate filaments, and are expressed in different types of epithelial cells. Within the liver, cytokeratin-19 is found primarily in ductal epithelial cells. Increased cytokeratin-19 expression, reflecting a more advanced ductular reaction, is associated with rapid fibrosis progression in cholestatic liver disorders [20]. Recent data reveal the mechanism of the ductular reaction involving biliary NF-kB-inducing kinase, which directly increases cholangiocyte proliferation and the secretion of proinflammatory cytokines, which stimulate liver macrophages and hepatic stellate cells, augmenting liver inflammation and fibrosis [21]. In laboratory diagnostics, some of cytokeratins are established noninvasive markers of carcinoma, such as CYFRA 21-1, measuring the soluble cytokeratin-19 fragment; tissue polypeptide antigen (TPA)—measuring the total of cytokeratin 8, 18 and 19; and tissue polypeptide specific antigen (TPS)—measuring soluble cytokeratin 18 fragments [22]. 

Although cytokeratin serum fragments have been used as established tumor markers, they also constitute a non-specific marker of tissue injury, with particular emphasis on CK-18, which represents a promising marker of non-malignant liver diseases. In the course of NAFLD, intermediate filaments are disassembled, with a simultaneous increase in monomeric cytokeratin synthesis and aggregation. Due to hepatocyte apoptosis, caspase cleaves cytokeratin-18 into stable fragments that are released into the circulation. The M65 ELISA detects total CK-18 serum levels, reflecting the extent of tissue damage, whereas the M30-Apoptosense test facilitates the assessment of the apoptotic cell death pathway [23,24]. Cytokeratin alterations leading to MDB formation in non-malignant liver disorders are complex and involve not only an imbalance in CK8/CK18 transcription levels, but also posttranslational modifications, such as ubiquitination, phosphorylation, transglutamination and sumoylation [24]. The determination of circulating levels of CK-18 contributes to a more accurate, non-invasive assessment of cell death, liver injury and hepatic fibrosis. Plasma cytokeratin-18 (CK-18) fragment levels correlate with the magnitude of hepatocyte apoptosis, liver inflammation, fibrosis and ballooning and were recently proposed to independently predict the presence of NASH [25]. Nevertheless, cytokeratin-18 fragments have modest accuracy for the diagnosis of NASH and a strong relationship with alanine transaminase (ALT) activity. As CK-18 is considered an accessory diagnostic and prognostic biomarker in acute and chronic liver diseases, especially MAFLD, it is important to understand the relationship of CK-18 M30 with various metabolic and inflammatory factors that are altered in obesity [22]. 

The aim of the study was to analyze the associations of cytokeratin-18 with not only fatty liver markers but also obesity-related metabolic disturbances, including insulin resistance, impaired lipid metabolism and the secretion of hepatokines, adipokines and pro-inflammatory cytokines.

## 2. Materials and Methods

### 2.1. Study Participants

The study involved subjects aged 25–65 years, women and men, obese (BMI 30–40 kg/m^2^) and overweight (BMI 25–29.9 kg/m^2^). Based on a medical examination and laboratory screening, only participants with no evidence of chronic diseases, particularly liver diseases, diabetes mellitus, severe dyslipidemias, endocrine disorders, cancer, chronic kidney disease, autoimmune diseases and inflammation, were included in the study. Exclusion criteria also included therapy with hormones, anti-inflammatory or lipid-lowering drugs, or other medicines/pharmaceuticals known to affect liver function, moderate to excessive use of alcohol, smoking, pregnancy or lactation. All recruited participants had a detailed medical examination and standard laboratory tests performed to evaluate health status and to exclude volunteers who met any exclusion criterion. 

The study was carried out in accordance with Declaration of Helsinki and with the Good Clinical Practice guidelines. The study was approved by the Bioethics Committee of the Jagiellonian University in Krakow (written consent, opinion No.1072.6120.56.2017), and all participants gave written informed consent. The study was conducted at the Department of Clinical Biochemistry, Jagiellonian University Medical College, Krakow, Poland.

### 2.2. Anthropometric Measures and Blood Collection

Anthropometric measurements included body weight, height, BMI, waist and hip circumferences and body fat content (Tanita Body Composition Analyser BC-418, Tanita, Tokyo, Japan). Blood pressure was measured in the supine position after 10′ of rest with an automatically inflating cuff. 

Participants were instructed to avoid strenuous exercise and alcohol consumption the day before blood collection. After a 12 h overnight fast, venous blood was collected and centrifuged (1000× *g* for 10 min at 4 °C within 30 min from collection), and serum and plasma samples were immediately frozen and stored at −80 °C for further analyses of glucose; insulin; the lipid profile; liver markers—cytokeratin-18 (CK-18), alanine aminotransferase (ALT), gamma-glutamyl transferase (GGT), fibroblast growth factor-19 (FGF-19), fibroblast growth factor-21 (FGF-21); adipokines—leptin, adiponectin, visfatin, resistin; and inflammatory markers—C-reactive protein (CRP), interleukin-6 (IL-6), soluble E-selectin (sE-selectin), soluble vascular cell adhesion protein-1 (sVCAM-1), monocyte chemoattractant protein-1 (MCP-1), soluble platelet/endothelial cell adhesion molecule -1 (sPECAM-1/CD31) and vascular endothelial growth factor (VEGF). 

An oral glucose tolerance test (OGTT) and high fat mixed meal tolerance test (HFMTT), both 5 time points, were performed on separate days. Venous blood samples—fasting, 30, 60, 90 and 120 min of OGTT—were collected in order to measure glucose and insulin, as well as fasting (before meal); 2, 4, 6 and 8 h of HFMTT were collected in order to measure postprandial TG concentrations. The detailed composition of the meal in the 8 h HFMTT was described previously [26]; in brief, the meal contained 73% fat, 16% protein and 11% carbohydrates, with a caloric value of 1018 kcal. 

### 2.3. Blood Analyses

Plasma glucose, total cholesterol, HDL-cholesterol and TGs were assayed through automated, enzymatic colorimetric methods (MaxMat SA, Montpellier, France). The intra and inter-assay variability coefficients were as follows: 2.3% and 3.5% (glucose), 1.4% and 3.4% (TGs), 1.4% and 3.8% (total cholesterol), 2.1% and 2.8% (HDL-cholesterol), respectively. LDL-cholesterol was calculated according to the Friedewald formula. The NEFA concentration was measured immediately in non-frozen serum using an enzymatic quantitative colorimetric method (Roche Diagnostics GmbH, Berlin, Germany).

Insulin was determined by performing an immunoradiometric method (DIAsource ImmunoAssays, Louvain-la-Neuve, Belgium) and read using a gamma counter (LKB Instruments, Victoria, Australia); within and between-run imprecision CVs were 2.1% and 6.5%, respectively. Homeostasis model assessment of insulin resistance (HOMA-IR index) was calculated as a ratio: [fasting glucose (mmol·L^−1^) × fasting insulin (µU·mL^−1^)]/22.5). 

Cytokeratin-18, that is soluble human intermediate filament protein fragments of cytokeratin 18, was determined with the M30 Apoptosense^®^ ELISA (PEVIVA^®^; VLVBio; Nacka, Sweden), which contains the M30 neo- epitope (K18Asp396-NE) released from human epithelial cells. According to the manufacturer’s instruction, M30 detects only caspase-cleaved fragments of cytokeratin-18 but not the native protein; thus, the presence of CK-18 in plasma reflects epithelial cell apoptosis. The limit of detection was 20 U/l. Activities of ALT and GGT were analyzed through routine diagnostics in a clinical laboratory. The assays for FGF-19 and FGF-21 employed the quantitative ELISAs (Human FGF-19 Quantikine ELISA, and Human FGF-21 Quantikine ELISA, respectively, R&D Systems Inc. Minneapolis, MN, USA). The limit of detection of human FGF-19 and FGF-21 immunoassays was 1.17 pg/mL and 4.67 pg/mL, the within-run coefficient of variation (CV) was 4.83% and 3.43% and the between-run CV was 5.13% and 7.5%, respectively. The fatty liver index (FLI) is based on a scoring algorithm involving the BMI, waist circumference, triglycerides and GGT and was calculated according to Bedogni et al. [8]. FLI = (e^0.953×loge (triglycerides) + 0.139×BMI + 0.718×loge (GTT) +0.053×waist circumference − 15.745^)/(1 + e^0.953×loge (triglycerides) + 0.139×BMI + 0.718×loge (GGT) + 0.053×waist circumference − 15.745^) × 100

Plasma leptin, adiponectin, resistin, IL-6, sE-selectin, MCP-1, sVCAM-1 and VEGF were determined via ELISA (R&D Systems Europe, LTD, Abington, UK). Within- and between-run imprecision CVs were 3% and 4% (leptin), 4% and 6% (adiponectin), 5.3% and 8.2% (resistin), 6% and 7% (IL-6), 6% and 8% (sE-Selectin), 5% and 6% (MCP-1), 3.5% and 7.7% (sVCAM-1) and 5.4% and 7.3% (VEGF), respectively. C-reactive protein was analyzed via a highly sensitive immunoturbidimetric method (APTEC Diagnostics, Sint-Niklaas, Belgium); the within-run imprecision CV was 1.66% and between-run imprecision CV was 2.08%. Visfatin (Nampt/PBEF), and PECAM-1/CD31 were measured via ELISA (BioVendor, Czech Republic); the inter- and intra-assay CVs were 6% and 7% (visfatin) and 1.7% and 7.4% (sPECAM-1), respectively.

### 2.4. Data Analysis

Nominal data were analyzed via a chi-square (χ^2^) test and presented as the number and percentage. The Shapiro-Wilk test was used to check for normal distribution. Continuous variables are presented as the median (Q1–Q3). Differences between the two studied groups were analyzed with the Mann-Whitney U-test. Areas under curves (AUCs) during the OGTT or HFMTT were calculated via the trapezoidal method. The Spearman rank correlations, the linear regression models and logistic regression models were used to find associations between variables. *p* values < 0.05 were considered significant. Statistical analyses were performed with Statistica software v.13.3 (StatSoft).

## 3. Results

### 3.1. Participant Characteristics

**Figure 1:**
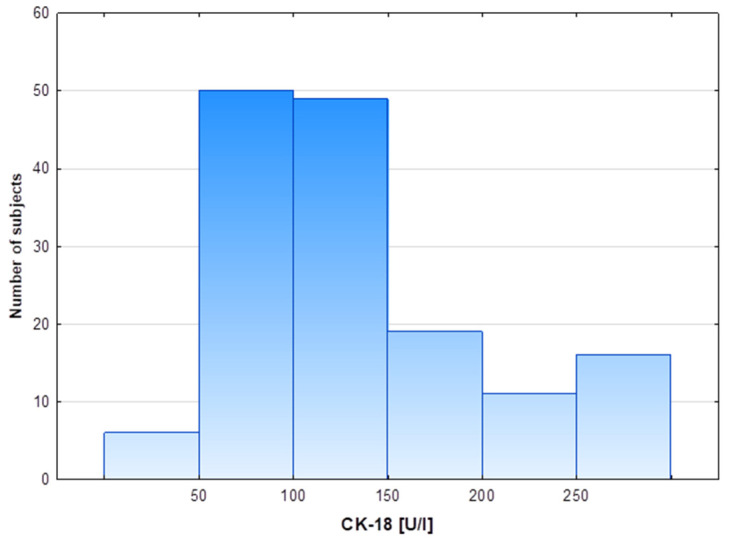
Distribution of cytokeratin-18 plasma levels in the study cohort.

The characteristics of the study population with excess body weight and different CK-18 plasma levels are presented in Table 1. Participants were assigned to groups depending on the CK-18 plasma level using 150 U/L as the cut-off point. The cut-off point was established based on data from a multicenter study that analyzed plasma CK-18, also with an M30-Apoptosense ELISA kit, in 150 healthy subjects with histologically excluded NASH through liver biopsy and for whom age, sex and BMI were similar to those of our cohort [27]. Moreover, distribution patterns of cytokeratin-18 plasma levels in the entire cohort showed that two thirds of subjects presented CK-18 values in the range of 50–150 U/L (Figure 1). Volunteers presenting relatively high CK-18 plasma levels constituted 30% of the entire study cohort and were similar in age to the group with lower CK values. The high CK-18 group differed from the low CK-18 group in regard to an increased proportion of the male sex and the severity of obesity, especially elevated indicators of abdominal obesity: waist circumference and WHR (Table 1). The two study groups differed significantly in body weight and BMI, but did not differ significantly in the percentage of body fat. The Q1 value of BMI in the low CK-18 group was below 30, so the value was within the BMI range indicating overweight and not obesity. Although the groups did not differ in the amount of body fat and the fat % even tended to be lower in the high CK-18 group, the distribution of adipose tissue within the waist indicates abdominal obesity in the high CK-18 group, which is associated with a worse metabolic phenotype. Despite no difference in fasting glycemia, patients with higher CK-18 showed elevated fasting insulin levels and HOMA-IR, as well as an increased area under the OGGT glucose and insulin concentration-time curves. For the high CK-18 group, the HOMA-IR Q2 and Q1 values were 3.50 and 2.65, respectively, pointing to the development of insulin resistance in these obese patients (Table 1). An analysis of the lipid profile indicated higher fasting and postprandial TG plasma levels in the high CK-18 subjects, whereas plasma total-CH, HDL-CH, LDL-CH and NEFAs were similar to those in obese patients with low CK-18 plasma levels (Table 1).

### 3.2. Liver and Inflammatory Markers

The mean CK-18 plasma concentration in the study population was 153.3 U/L, the distribution pattern of the values was non-normal, the median (Q1–Q3) values were 118.6 (87.6–156.7), respectively, the minimum CK-18 value observed was 15.6 U/L and the maximum values was 801.7 U/L. 

The distant ranges of CK-18 values in both groups were accompanied by different levels of classic liver markers; ALT activity and GGT activity were significantly higher in the high CK-18 group than in the low CK-18 group (Table 2). A similar difference was found with the FLI, as well with the hepatokine FGF-21, whereas the FGF-19 plasma level did not differ between groups. Serum concentrations of adipokines, leptin, adiponectin, visfatin and resistin and inflammatory markers associated with low-grade inflammation in obesity, CRP, IL-6, sE-selectin, sVCAM, sPECAM and VEGF, were similar in both groups, regardless of CK-18. The only inflammatory marker elevated in the high CK-18 group was MCP-1 (Table 2).

### 3.3. Associations of CK-18 with Anthropometric and Metabolic Markers of Obesity

**Figure 2:**
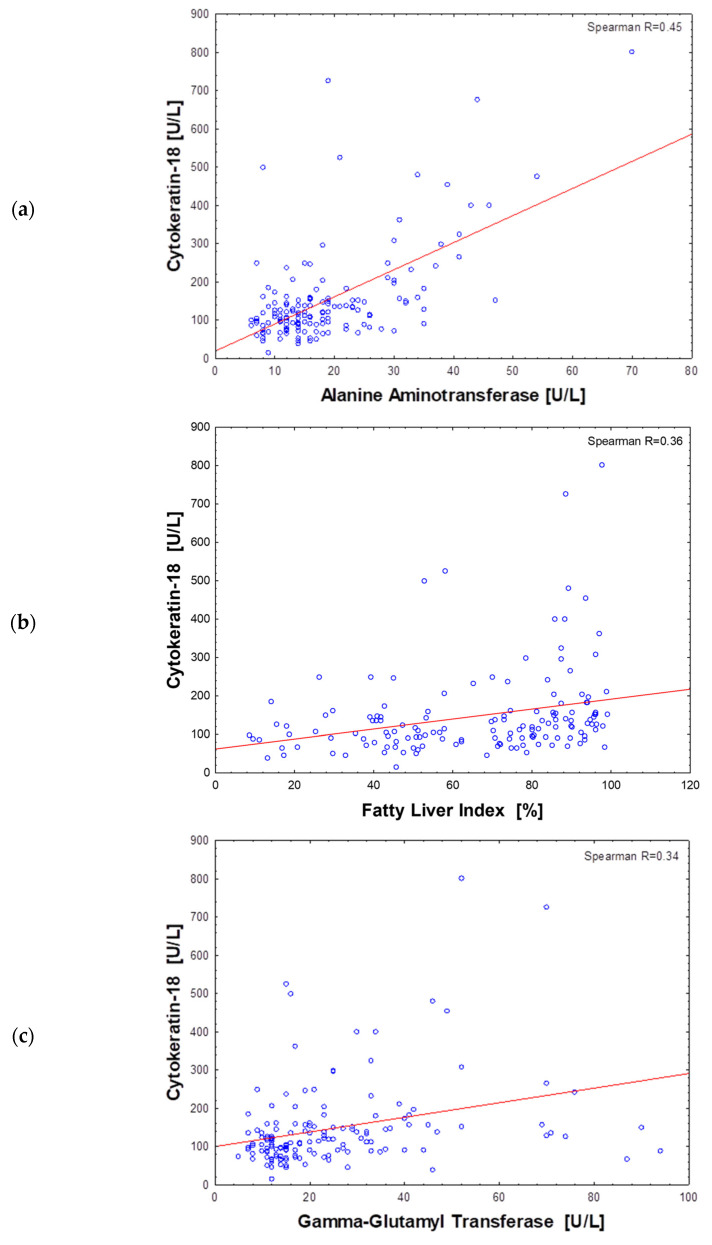
Scatter plot of the cytokeratin-18 plasma level and alanine aminotransferase (**a**), fatty liver index (**b**) and gamma-glutamyl transferase (**c**) activity in all subjects (*n* = 151).

The analysis of Spearman rank correlations found the association of CK-18 plasma levels with some anthropometric and metabolic features of obesity and its complications. The strongest correlations of CK-18 were observed for liver markers: ALT activity (R = 0.45), FLI (R = 0.36), GGT (R = 0.34) (Figure 2) and for visceral adiposity indicators: waist circumference (R = 0.33), body weight (R = 0.33) and WHR (R = 0.30). All other variables analyzed were less correlated with CK-18 (R < 0.30) (Table 3). Nevertheless, the significant correlations of CK-18 with fasting insulin and glucose-induced insulin output were found. Similarly, fasting and postprandial TG plasma levels positively correlated with CK-18. A weak but significant correlation was also observed for FGF-21, for which secretion and action in obesity are often disturbed. The above results indicated the relationship of elevated CK-18 with insulin resistance in obesity. In the entire study cohort, the only statistically significant negative relationship was observed for CK-18 and adiponectin, which was in line with a positive association with pro-inflammatory MCP-1 (Table 3). 

**Figure 3:**
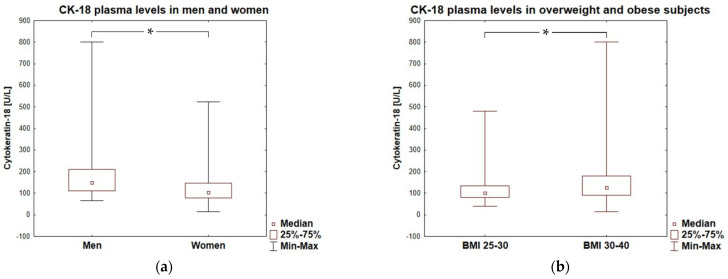
Cytokeratin-18 plasma levels in men and women (**a**) and in overweight and obese subjects (**b**). * represent a significant difference (*p* < 0.05).

In the entire cohort, 27 subjects (18%), crossed the level of CK-18 > 200 U/L which is (according to *M30 Apoptosense^®^ ELISA* assay manufacturer, PEVIVA) the cut-off point indicative for substantial liver disease (for example NASH), if the presence of an epithelial carcinoma is excluded. Such high levels of CK-18 were observed in 26% of the men and 15% of women enrolled in the study. Men presented higher values of cytokeratin-18 than women, with an average of 149 (111–210) U/L *vs.* 105 (78–148), *p* = 0.0002, respectively (Figure 3a). Moreover, CK-18 plasma levels > 150 U/L referred to half of men (*n* = 21/42; 50%) and ca. a quarter of women (*n* = 25/109; 23%) tested in our study, which was a significant difference in frequency (*p* = 0.0012). The plasma concentration of CK-18 not only correlated with BMI, but also significantly differed between overweight and obese subjects, being lower in the participants with a BMI < 30 [100.5 (80.5–134.0) U/L] compared to that in participants with a BMI ≥ 30 [126.0 (90.3–180.3) U/L; *p* = 0.0130]—Figure 3b. The frequency of CK-18 values > 150 U/L was significantly higher in obese patients then in overweight subjects (36% *vs.* 14%; *p* = 0.0065).

To examine, in detail, the association of plasma CK-18 with biomarkers of obesity-related metabolic disturbances, linear regression models were tested (Table 4). After an adjustment for sex and age (model 1) or for sex, age and BMI (model 2), only ALT activity was found as a factor independently influencing CK-18. In all regression models, VIF was low (VIF < 2), indicating that there was no multicollinearity. Moreover, ALT was a predictor for elevated (>150 U/L) CK-18 in separate logistic regression models adjusted for sex and age or sex, age and BMI (Table 5). 

## 4. Discussion

In the presented study, the cytokeratin-18 fragment plasma level was elevated in obese compared to overweight patients and was strongly associated with liver injury markers, such as ALT, FLI and GGT, as well as with indexes of abdominal obesity. Linear regression, as well as logistic regression models, indicated that after an adjustment for sex, age and BMI, the independent predictor of high CK-18 was ALT activity. Other studies confirmed the elevated CK-18 fragment levels in obese subjects, also reporting the reversibility of this trend upon diet-induced weight loss, physical exercise and after bariatric surgery [28,29,30]. In our study of obese subjects, the average concentration of CK-18 fragments was 118.6 U/L (87.5–156.7), which was very similar to the average value of 118.5 U/L (87.2–188.9) reported from a Chinese cohort with NAFLD [31]. Moreover, the CK-18 concentration after the remission of NAFLD decreased to 84.7 U/L (53.3–135.8), thus reaching a range of values similar to that in the low CK-18 group in our study on the Polish population. It was reported that the obesity state modifies (increases) the association between heavy metal exposure and serum liver injury biomarkers, including CK-18 fragments [32]. The degree of obesity was also found to affect the performance of liver fibrosis biomarkers. In obese patients, the best predictor of liver fibrosis was MACK-3, which involved CK-18 fragments, as well as FIB-4, which were not affected by BMI [33]. Contrary to this, the widely used NAFLD Fibrosis Score loses specificity in obese individuals and probably requires BMI-adjusted cutoffs [33].

The strong correlations of CK-18 with ALT were also observed in other studies, and changes in CK-18 M65 levels after the 12 weeks of exercise were significantly correlated with changes in alanine aminotransferase activity (r = 0.62) [29]. A study on a population of 688 subjects showed, by regression analysis, that the total CK-18 (M65) plasma levels positively correlated with cardiometabolic disorder components, including obesity, blood pressure, glycated hemoglobin A1c, ALT and HDL [34]. In this Chinese population, total CK-18 levels were significantly increased in subjects with multiple cardiometabolic factors and were an independent risk factor for cardiometabolic disorders after accounting for general cardiometabolic risk parameters or the presence of NAFLD. Another study showed that decreases in serum levels of CK-18 were strongly associated not only with decreases in ALT but also with improved liver histology in adults or children with NAFLD. The reductions in CK18 did not perform better than the alanine aminotransferase level in identifying histologic changes in NAFLD [35]. However, the levels of traditional biomarkers, such as ALT and AST, are sometimes elevated without the presence of hepatocellular injury; thus, only significant fold increases in ALT (>3–5 times URL) are considered adverse and indicative of hepatocellular injury. In this context, it is suggested that CK-18 could serve as an early marker of liver apoptosis and drug-induced liver injury. Furthermore, CK-18 is less affected by muscle mass and activity than ALT and AST, with only a 0.9-fold-change observed in its expression after exercise, compared to a 2.5–5.5-fold-increase in ALT and AST, respectively [36]. In a study of obese children, linear regression showed that CK-18 was associated with WHR, FLI and adiponectin levels, which is consistent with the results of the correlation analysis in our study. Among other candidate markers studied (glutamate dehydrogenase, macrophage colony stimulating factor receptor and osteopontin), CK-18 is more sensitive and specific in diagnosing early-stage drug-induced liver injury and has a demonstrated strong relationship with hepatocellular injury in the clinic [37]. Recent studies also indicated the association of CK-18 with molecular markers of liver diseases, such as various types of microRNA, in particular miRNA-122 [36,38,39,40]. The combination of CK-18 with three microRNAs, miR-122, miR-192 and miR-21, gave the AUROC of 0.83 for diagnosing NASH [16]. However, these molecular markers have no clinical application yet.

Different cut-offs of CK-18 fragment plasma levels have been chosen in different studies on NASH prediction markers. In a meta-analysis of 15 studies reporting concentrations of CK-18 (M30), the cut-offs ranged from 122 to 380 U/L and the combined sensitivity and specificity were 0.75 and 0.77, whereas the AUROC value of the CK-18 testing in predicting NASH was 0.82, showing the ability to distinguish steatohepatitis from simply steatosis [41]. In a group of sixty overweight and obese patients with confirmed liver fibrosis, baseline serum CK-18 levels were 290 ± 98 U/L and decreased significantly over a 6-month period upon a caloric restriction diet to 217 ± 64 [28]

These values were higher than those reported in our study, which is consistent with the fact that participants involved in our study were obese but clinically healthy. Nevertheless, it is worth noting that CK-18 is released excessively from the liver into the circulation not only in NAFLD/NASH, but also in alcoholic liver disease (ALD), hepatitis B and hepatitis C patients and drug-induced liver injury [37,38,42,43,44]. Additionally, in acetaminophen-induced acute liver failure, serum CK-18 levels were associated with liver dysfunction and post-transplant graft failure, with levels > 900 U/L indicating 1-year graft loss [45]. In a study by Feldstein et al., with a cohort consisting of 139 patients with biopsy-proven NAFLD from eight centers across the United States, CK-18 fragments ranged from 68 to 3000 U/L and were markedly increased in patients with NASH as compared to levels in those without NAFL (median (Q1, Q3): 335 (196, 511) vs 194 (151, 270) U/L) [27]. In contrast, the CK-18 values observed in our obese cohort were significantly lower (119, (87–157) U/L) and more in line with the values determined for 150 age-matched healthy controls in the US study, 145 (126, 190) U/L. This CK-18 median value of 145 in healthy volunteers was the exact 66th percentile of CK-18 in our cohort and was approximately the basis for proposing the cut-off point for low and high CK-18 groups in our study. 

The results of our study are part of the trend of recognizing the process of fatty liver and hepatitis in obesity as MAFLD. Not only elevated fasting glycemia and insulin and HOMA-IR, but also higher glucose-stimulated insulin output, characterized patients with high CK-18 fragment levels. Given the accompanying fasting and postprandial hypertriglyceridemia, we can clearly conclude that insulin resistance was a metabolic complication of obesity associated with high circulating CK-18. Similar conclusions were recently drawn by Lopez-Sanchez (2022) indicating that circulating CK-18 levels were elevated in patients with MAFLD and additionally correlated with specific microRNAs. 

In another study, the HOMA-IR, an index of insulin resistance, was included together with the plasma level of CK-18 and AST activity into the MACK-3 index, being predictive of fibrotic non-alcoholic steatohepatitis [38]. Treating hypertriglyceridemia helps to prevent hepatocyte injury partially through a mechanism involving cytokeratins. The effect was confirmed in a mouse model in which the use of a PPARα agonist, fenofibrate, inhibited the disruption of intermediate filaments, further reducing inflammation and improved the phenotype [46]. In an animal model of fructose-induced metabolic syndrome, the associations between serum CK-18 levels and the degree of liver damage and insulin resistance were reported [47]. One of the mechanisms of glucose toxicity in hyperglycemia is increased O-GlcNAcylation. Increased O-GlcNAcylated proteins may be associated with hepatic histological morbidities [48]. In the liver, transcriptional factors, FoxO1, PGC-1α, CRTC2, LXR and ChREBP, are found to be regulated by O-GlcNAcylation, leading to enhanced expression of gluconeogenic and lipogenic genes. Hyperglycemia induces elevated O-GlcNAcylation of these key transcription factors and cofactors, promoting gluconeogenesis and lipogenesis, which further diminishes insulin sensitivity [49]. Interestingly, the process of glycosylation applies also to epithelial cytokeratins, being able to modify the properties of the cytokeratin network [50]. Convincing data from an animal model show that abolished glycosylation of CK-18 leads to hyperglycosylation-induced epithelial tissue damage [51]. CK-18 glycosylation was found to promote a phospho-Akt active state that inhibits cell death in the liver. Therefore, CK-18 appears to serve as a cytoprotective and anti-apoptosis buffer during conditions that promote increased protein O-GlcNAcylation [46].

There are also reports of other cytoskeletal intermediate filaments being associated with obesity and its metabolic complications. Cytokeratin-7 (CK-7) is an intermediate filament expressed in the simple epithelium and was recently found to be up-regulated in endocrine islets of Langerhans in experimental diabetes [52]. Interestingly, the same study showed that CK-7 filament formation in islets is dependent on the presence and levels of CK-18, suggesting the potential contribution of pancreatic CK-18 to streptozotocin-induced diabetes. Cytokeratins in epithelial cells form heterodimers, consisting of different type I (e.g., CK-18, CK-19, CK-20) and type II (e.g., CK-7, CK-8) polypeptides, and hepatocytes are unique in this regard as they contain the CK-8/CK-18 pair only [24]. There are also type III intermediate filaments proteins, such as vimentin, glial fibrillary acidic protein, desmin and peripherin [20]. Park et al. provided well-documented evidence that vimentin, like cytokeratins, is cleaved by multiple caspases during apoptosis [53]. The proteomic analysis of the obesity-induced mouse liver revealed that a high-fat diet changed the expression of intermediate microfilament cytoskeleton proteins, in particular up-regulating CK-18, CK-8 and vimentin [53]. Recently, a study on vimentin-null mice with high-fat diet-induced obesity showed less weight gain, less adiposity, improved glucose tolerance and lower fasting glucose, as well as higher levels of triglycerides and free fatty acids [54]. The mechanism of action of vimentin was mediated by the expression of CD36 and the glucose transporter GLUT4 on the cell membrane of adipocytes, linking intermediate filaments and intracellular trafficking with obesity and insulin resistance. Other studies support the hypothesis that the intermediate filament protein vimentin is involved in obesity and type 2 diabetes, by participating not only in GLUT4 vesicle trafficking but also in lipolysis through an interaction with hormone-sensitive lipase [55,56]. Thus, vimentin, like CK-18, seems to be a promising marker for predicting obesity and liver disease, although its significance requires further research. 

In our study, elevated CK-18 plasma levels were accompanied by elevated FGF-21 levels in obesity, whereas FGF-19 was not related. FGF-21 is a liver-secreted hormone, playing important roles in regulating energy balance and glucose and lipid homeostasis. The elevated FGF-21 plasma levels in obesity possibly reflect obesity-related FGF21 resistance and the action of endogenous FGF21 inactivation enzymes. As for today, several FGF21 analogues and mimetics have progressed to clinical trials in patients with obesity, type 2 diabetes mellitus and NASH. Although the primary end points of glycemic control have not been met, substantial improvements were observed with dyslipidemia, hepatic fat fractions and serum markers of liver fibrosis in patients with NASH [57]. Data on the simultaneous testing of CK-18, FGF19 and FGF21 are scarce; however, there are few reports of results similar to the presented ones. In a prospective study of a Chinese population, both CK-18 and FGF21 were elevated in NAFLD, though baseline FGF21, but not the CK18 level, was an independent predictor for the development of simple steatosis. NAFLD patients who had remission during follow-up had significantly lower baseline M30 levels than those who sustained NAFLD [31]. A correlation of FGF-21 and CK-18 was also observed in another study, although with regards to full-length CK-18 (M65), not the caspase cleaved form M30 [29]. In this Japanese study, 12 weeks of physical exercise reduced CK18 and FGF21 levels in patients with NAFLD, despite no changes in the body mass index, indicating that the exercise-induced metabolic switch and proper metabolic flexibility is probably more related to the normalization of FGF-21 and CK-18 than simply a reduction in body weight. On the other hand, a shorter (4-week) exercise training intervention did not reduce either ALT or CK-18 despite decreasing postprandial insulin [58]. Similarly, in a study involving diabetic type 2 patients, serum levels of both FGF-21 and CK-18 were not evidently associated with the presence of NAFLD or liver fibrosis [59].

In a meta-analysis by He et.al. (2017) of 12 studies, the plasma concentration of FGF-21 in NAFLD was significantly higher than that in a control group, and the diagnostic odds ratio, sensitivity and specificity of FGF-21 testing for NASH were 5.70, 0.62 and 0.78, respectively [41]. Moreover, combining two or more individual biomarkers as a panel could obtain a better predictive value for NASH, and in a design including CK-18 and FGF-21, the AUROC was 0.95 compared to the CK-18 or FGF-21 assay, with the combined (from seven studies) sensitivity and specificity being 0.92 and 0.85, respectively [41]. According to current EASL guidelines, the FIB-4 index is clinically useful and cost-effective, though it is mainly the tool for excluding, not confirming, patients with advanced liver fibrosis [60]. In addition, other tools, such as transient elastography, are limited to specialized centers. No highly sensitive and specific tests are available to differentiate simple steatosis from steatohepatitis [61]. The approach focused on combining biomarkers seems to be more useful, but requires prior research on potential markers and the interrelationship between them, and the results of our study can also contribute to this.

FGF-21 agonism has been shown to reverse hepatic fat infiltration and improve insulin sensitivity by increasing fatty acid oxidation and glucose uptake by adipocytes [62]. However, as in the pathogenesis of steatohepatitis and liver fibrosis different pathways are dysregulated, combination therapy with an FGF21 analog along with inflammatory pathway inhibition are of interest. Recently, Puengel et al. revealed that combined therapy with a CCR2/CCR5 antagonist, which blocks the receptor for MCP-1, and an FGF-21 analogue synergizes in ameliorating steatohepatitis and fibrosis in mice subjected to dietary models of NASH and fibrosis [63]. The same authors provided data on the correlation of circulating FGF-21 with CK-18 (fragment M30), as well as with GGT and ALT. In this study, involving 85 patients with biopsy-confirmed NAFLD, MCP-1 serum levels were significantly elevated in patients with advanced fibrosis compared to those in patients without, based on histopathology, as well as the FIB-4 score; however, the MCP-1 levels were not associated with NASH activity [63]. While the average values of ALT (36 U/L in NAFL and 46 U/L in NASH) and GGT (29 in NAFL and 50 in NASH) in this study were approximately twice as high as those in our cohort (ALT 16 U/L, GGT 19 U/L), the median MCP-1 levels were comparable, being 342 pg/mL in NAFL and 350 pg/mL in NASH, compared to 338 pg/mL in our obese subjects. The median of MCP-1 plasma levels in the high CK-18 group (351.5 pg/mL) in our study were significantly higher than those in the low CK-18 group (330.5 pg/mL), but were comparable to those in NASH patients (350 pg/mL). Although serum concentrations of most of the investigated markers of inflammation, including CRP and IL-6, did not differ between the high and low CK-18 groups, a significantly elevated plasma level of MCP-1 probably indicates an ongoing low-grade chronic inflammatory process. We cannot identify the source of this cytokine; it may be of adipose tissue or hepatic stellate cell origin, known to be a versatile source of soluble immunological factors, including cytokines [64]. During obesity, hypertrophic adipocytes release proinflammatory adipokines and cytokines, such as leptin, visfatin, resistin, MCP-1 and IL-6, which contribute not only to local but also systemic and liver inflammation [65]. The lack of a relationship of CK-18 and IL-6 plasma concentrations, measured in our subjects, may be due to the complex action of this cytokine in the liver. A study using a mouse NASH model revealed that the blockade of IL-6 signaling enhanced hepatic steatosis but improved liver injury [66]. Dual IL-6 action was found to be mediated by transcription factors—decreased sterol regulatory binding protein-1 (SREBP1) and increased peroxisome proliferator-activated receptor-alpha—on the one hand, and by STAT3 activation, leading to inflammation and liver damage, on the other hand.

Other studies indicated the association of CK-18 fragments (M30) with inflammation, contrary to full-length CK (M65), which was not associated with inflammation [67]. In the study on the effects of exercise on liver metabolism in alcohol drinkers, the decrease in CK levels was not accompanied by an improvement in inflammatory parameters [68]. Although inflammation is a major feature of steatohepatitis, CK-18 is a marker of apoptosis and does not necessarily reflect the severity of inflammation. Nevertheless, there are some data on the molecular mechanism by which CK-18 is involved in cell death, including the regulation of apoptotic genes FAS and FADD, as well as immune genes CxCL2 and CD79B, on the transcriptional level [69].

The main limitation of our study was the lack of elastography measurements of the liver or imaging tests on the basis of which the morphological parameters of the liver and the risk of MAFLD could be assessed. Nevertheless, we have made efforts to evaluate many classical and new laboratory parameters that can indirectly assess the liver and the risk factors of liver disease in obesity. Moreover, the gold standard in the diagnosis of fatty liver/fibrosis is histological evaluation of liver biopsies, which could not be used as it is an invasive method. It is also suggested in some studies that it is better to detect both CK-18 fragments with the M30 antibody and full-length cytokeratin 18 with the M65 antibody and calculate the ratio, which allows for an assessment of the relative levels of apoptotic to necrotic processes [31]. However, most of the studies indicate that both the higher amount of M30 and the higher ratio of M30 to M65 are each correlated with the liver disease score and apoptosis. In conjunction with other markers, the CK-18 fragment level was proposed as a biomarker for fibrotic NASH diagnosis, for which quantification provides the most used biomarker of steatohepatitis, despite its low accuracy [20,38,70].

Based on the study of 151 subjects with excess body weight, without clinical symptoms or laboratory markers of metabolic diseases, we can conclude that the plasma CK-18 M30 cut-off point at 150 U/L allows to distinguish between two metabolic phenotypes in obesity. Cytokeratin-18 fragment plasma levels above 150 U/L may reflect even mild liver injury and involve metabolic complications of obesity, especially insulin resistance, disturbed lipid postprandial metabolism, low-grade inflammation and inefficient FGF21 signaling.

## Figures and Tables

**Table 1:** Characteristics of subjects included in the study: comparison of groups according to the cytokeratin-18 plasma level.

	All *n* = 151	Low CK-18 *n* = 105	High CK-18 *n* = 46	*p*-Value
Age	47	49	46	
[years]	(39–57)	(39–58)	(38–57)	0.2934
Sex	109	84	25	
[% female]	(72%)	(80%)	(54%)	0.0012
Anthropometrics				
Body weight	91.5	88	100.6	
[kg]	(81–103.9)	(79–99)	(86.8–111.1)	0.0013
BMI	32.45	32.01	33.83	
[kg/m^2^]	(30.02–35.30)	(29.31–35.09)	(31.68–35.69)	0.0134
Body fat	38.30	38.9	35.4	
[%]	(32.90–42.20)	(33.9–42.6)	(31.25–41.33)	0.1006
Waist circumference	102.0	99.5	110.0	
[cm]	(95.0–111.5)	(93.8–108.0)	(99.5–118.0)	0.0005
WHR	0.89	0.85	0.96	
	(0.82–0.97)	(0.82–0.92)	(0.87–1.01)	0.0004
Systolic BP	129	125	130	
[mmHg]	(120–140)	(120–132)	(120–142)	0.0591
Diastolic BP	84	82	87.5	
[mmHg]	(80–90)	(80–90)	(80–90)	0.3284
Glucose metabolism				
Glucose	5.15	5.10	5.30	
[mmol/L]	(4.80–5.60)	(4.80–5.53)	(4.90–5.70)	0.0724
Glucose AUC OGTT	3474	3342	3891	
[mmol/L·min]	(2922–4188)	(2919–3855)	(3372–4415)	0.0078
Insulin	13.05	12.35	16.10	
[mU/L]	(9.70–19.10)	(9.58–16.58)	(12.05–24.83)	0.0078
Insulin AUC OGTT	39462	35724	45498	
[mmol/L·min]	(27138–60696)	(25473–55901)	(32247–78528)	0.0138
HOMA-IR	2.94	2.81	3.50	
	(2.20–4.56)	(2.15–3.92)	(2.65–6.08)	0.0194
Lipid metabolism				
Total cholesterol	5.39	5.34	5.57	
[mmol/L]	(4.80–6.15)	(4.82–5.99)	(4.79–6.34)	0.3683
HDL cholesterol	1.29	1.29	1.28	
[mmol/L]	(1.13–1.46)	(1.14–1.46)	(1.12–1.46)	0.5375
LDL cholesterol	3.45	3.42	3.65	
[mmol/L]	(2.90–4.14)	(2.96–4.07)	(2.70–4.44)	0.5463
Fasting triglycerides	1.31	1.21	1.48	
[mmol/L]	(0.95–1.78)	(0.89–1.68)	(1.21–2.25)	0.0096
Postprandial triglycerides AUC	3482	3216	3900	
[mmol/L·min]	(2494–4884)	(2314–4573)	(3356–5291)	0.009
Fasting NEFAs	0.69	0.67	0.79	
[mmol/L]	(0.53–0.97)	(0.51–0.94)	(0.59–1.03)	0.0678

Data presented as the median with quartiles (Q1–Q3) and compared between groups by using the Mann-Whitney U test. Categorical variables were analyzed via Pearson’s chi-square test. Low CK-18 -group with fasting cytokeratin-18 plasma level ≤ 150 U/L; high CK-18 group with cytokeratin-18 plasma levels > 150 U/L; BMI—body mass Index, WHR—waist-to-hip ratio, AUC OGTT—area under curve during oral glucose tolerance test; postprandial triglycerides AUC—area under curve for triglyceride plasma concentration during 8 h long mixed meal tolerance test; NEFAs—non esterified fatty acids.

**Table 2:** Liver and inflammatory markers in groups according to the cytokeratin-18 plasma level.

	All *n* = 151	LOW CK-18 *n* = 105	HIGH CK-18 *n* = 46	*p*
Liver markers				
CK-18 [U/L]	118.6 (87.5–156.7)	95.6 (73.8–121.03)	220.6 (160.7–322.9)	<0.0001
ALT [U/L]	16.0 (12.0–23.0)	14.0 (11.0–19.0)	23.5 (16.0–34.8)	<0.0001
GGT [U/L]	19.0 (12.5–32.0)	16.0 (12.0–27.0)	25.0 (17.0–41.5)	0.0004
FLI	73.01 (45.49–87.54)	62.27 (43.31–81.83)	86.78 (66.42–93.84)	0.0001
FGF-19 [pg/mL]	112.60 (69.64–188.36)	112.60 (69.64–185.19)	106.36 (71.22–207.57)	0.8556
FGF-21 [pg/mL]	212.83 (127.83–303.99)	191.29 (119.74–273.04)	263.17 (148.91–387.26)	0.0231
Adipokines				
Leptin [pg/mL]	30,726 (19,740–46,988)	32,190.2 (21,683–50,153)	27,307.35 (17,732–41,835)	0.3091
Adiponectin [ng/mL]	6251 (4293–8899)	6533 (4376–9614)	5542 (3693–7462)	0.0518
Visfatin [ng/mL]	0.90 (0.60–1.35)	0.89 (0.60–1.26)	1.03 (0.63–1.48)	0.2613
Resistin [ng/mL]	9.20 (7.39–11.67)	9.5 (7.44–11.73)	8.74 (7.53–11.15)	0.4401
Inflammatory markers				
CRP [mg/L]	1.60 (0.68–3.88)	1.60 (0.64–3.79)	1.69 (0.84–3.89)	0.6588
IL-6 [pg/mL]	1.24 (0.87–1.74)	1.28 (0.86–1.89)	1.11 (0.88–1.56)	0.4645
sE-Selectin [ng/mL]	39.02 (29.79–48.98)	38.1 (29.7–47.45)	40.65 (31.92–52.42)	0.1751
sVCAM [ng/mL]	599.22 (511.22–711.46)	584.37 (520.41–700.75)	619.13 (512.79–749.47)	
MCP-1 [pg/mL]	338.37 (271.31–415.88)	330.55 (257.63–400.42)	351.54 (308.94–421.96)	0.0469
sPECAM [ng/mL]	70.10 (59.95–84.77)	70.90 (61.15–84.66)	68.50 (56.48–86.07)	0.4071
VEGF [pg/mL]	312.7 (200.2–501.7)	348.4 (206.8–529.6)	276.1 (189.1–465.9)	0.1736

Data presented as median with quartiles and compared between groups by using the Mann-Whitney U test. Categorical variables were analyzed via Pearson’s chi-square test. Low CK-18, group with fasting cytokeratin-18 plasma level ≤ 150 U/L; high CK-18, group with cytokeratin-18 plasma levels > 150 U/L; ALT—alanine transferase, FLI—fatty liver index.

**Table 3:** Spearman rank correlation between cytokeratin-18 plasma levels and anthropometrics, liver markers and metabolic variables in the entire group of subjects participating in the study (*n* = 151).

	R	*p*
ALT	0.45	<0.0001
FLI	0.36	<0.0001
GGT	0.34	<0.0001
Waist circumference	0.33	<0.0001
Body weight	0.33	<0.0001
WHR	0.30	0.0005
BMI	0.23	0.0055
Fasting insulin	0.25	0.0036
Insulin AUC OGTT	0.23	0.0079
Fasting glucose	0.16	0.0472
Glucose AUC OGTT	0.23	0.0058
Fasting TG	0.16	0.0489
Postprandial TG	0.20	0.0126
FGF-21	0.18	0.0245
Adiponectin	−0.23	0.0049
MCP1	0.20	0.0209

Statistically significant correlations: *p* < 0.05; R-Spearman coefficient; ALT indicates alanine aminotransferase; FLI, fatty liver index; GGT, gamma-glutamyl transferase; WHR, waist-to-hip ratio; BMI, body mass index; AUC indicates area under the curve; OGTT, oral glucose tolerance test; MCP1, monocyte chemoattractant protein 1.

**Table 4:** Linear regression models of the association between the cytokeratin-18 plasma level and obesity-related biomarkers.

	Model 1	Model 2
	β (95% CI)	β (95% CI)
ALT	0.41 (0.20–0.61)	0.40 (0.19–0.61)
Insulin AUC OGTT	0.13 (−0.06–0.32)	0.12 (−0.08–0.32)
Fasting triglycerides	−0.11 (−0.29–0.08)	−0.11 (−0.29–0.08)
MCP1	0.05 (−0.14–0.23)	0.05 (−0.14–2.23)
Adiponectin	−0.08 (−0.28–0.12)	−0.09 (−0.29–0.12)

Model 1 adjusted for sex and age (*n* = 151); model 2 adjusted for sex, age and BMI (*n* = 151). Abbreviations: ALT—alanine aminotransferase; insulin AUC OGTT—area under the curve of the insulin concentration during the oral glucose tolerance test; MCP1—monocyte chemoattractant protein 1.

**Table 5:** Contribution of obesity-related biomarkers (odds ratio and 95% confidence interval (CI)) to the risk of elevated (>150 U/L) cytokeratin plasma level.

	Model 1	*p*	Model 2	*p*
	β (95% CI)		β (95% CI)	
ALT	1.13 (1.05–1.22)	0.0017	1.13 (1.04–1.22)	0.0024
Insulin AUC OGTT	1.00 (1.00–1.00)	0.2891	1.00 (1.00–1.00)	0.4851
Fasting triglycerides	0.99 (0.51–1.91)	0.9744	0.98 (0.5–1.89)	0.9425
Fasting NEFA	0.39 (0.07–2.12)	0.2733	0.35 (0.06–1.98)	0.2354
MCP1	1.00 (1.00–1.01)	0.5877	1.00 (1.00–1.01)	0.6866

Logistic regression model 1 adjusted for sex and age (*n* = 151); model 2 adjusted for sex, age and BMI (*n* = 151). Abbreviations: ALT—alanine aminotransferase; insulin AUC OGTT—area under curve of insulin concentration during oral glucose tolerance test; NEFA—non-esterified fatty acids; MCP1—monocyte chemoattractant protein 1.

## Data Availability

Not applicable.
